# Comprehensive characterization of elevated tau PET signal in the absence of amyloid-beta

**DOI:** 10.1093/braincomms/fcac272

**Published:** 2022-10-26

**Authors:** Alexandra J Weigand, Lauren Edwards, Kelsey R Thomas, Katherine J Bangen, Mark W Bondi, for the Alzheimer’s Disease Neuroimaging Initiative

**Affiliations:** Joint Doctoral Program in Clinical Psychology, San Diego State University/University of California San Diego, San Diego, CA 92120, USA; Joint Doctoral Program in Clinical Psychology, San Diego State University/University of California San Diego, San Diego, CA 92120, USA; VA San Diego Healthcare System, San Diego, CA 92161, USA; Department of Psychiatry, University of California San Diego, San Diego, CA 92093, USA; VA San Diego Healthcare System, San Diego, CA 92161, USA; Department of Psychiatry, University of California San Diego, San Diego, CA 92093, USA; VA San Diego Healthcare System, San Diego, CA 92161, USA; Department of Psychiatry, University of California San Diego, San Diego, CA 92093, USA

**Keywords:** Alzheimer’s disease, primary age-related tauopathy, biomarkers, amyloid-beta, tau

## Abstract

Recently proposed biomarker-only diagnostic frameworks propose that amyloid-beta is necessary for placement on the Alzheimer’s disease continuum, whereas tau in the absence of amyloid-beta is considered to be a non-Alzheimer’s disease pathologic change. Similarly, the pathologic designation of tau in the absence of amyloid-beta is characterized as primary age-related tauopathy and separable from Alzheimer’s disease. Our study sought to identify an early-to-moderate tau stage with minimal amyloid-beta using PET imaging and characterize these individuals in terms of clinical, cognitive and biological features. Seven hundred and three participants from the Alzheimer’s Disease Neuroimaging Initiative were classified into one of the four groups (A−/T−, A−/T+, A+/T− and A+/T+) based on PET positivity or negativity for cortical amyloid-beta (A−/A+) and early-to-moderate stage (i.e. meta-temporal) tau (T−/T+). These groups were then compared on demographic and clinical features, vascular risk, multi-domain neuropsychological performance, multi-domain subjective cognitive complaints, apolipoprotein E epsilon-4 carrier status and cortical thickness across Alzheimer’s disease-vulnerable regions. The proportion of participants classified in each group was as follows: 47.23% A−/T−, 13.51% A−/T+, 12.23% A+/T− and 27.03% A+/T+. Results indicated that the A−/T+ and A+/T+ groups did not statistically differ on age, sex, depression levels, vascular risk and cortical thickness across temporal and parietal regions. Additionally, both A−/T+ and A+/T+ groups showed significant associations between memory performance and cortical thickness of temporal regions. Despite the different pathologic terminology used for A−/T+ and A+/T+, these groups did not statistically differ on a number of clinical, cognitive and biomarker features. Although it remains unclear whether A−/T+ reflects a pathologic construct separable from Alzheimer’s disease, our results provide evidence that this group typically characterized as ‘non-Alzheimer’s pathologic change’ or ‘primary age-related tauopathy’ should be given increased attention, given some similarities in cognitive and biomarker characteristics to groups traditionally considered to be on the Alzheimer’s continuum.

## Teaser

Weigand *et al*. report that a group of older adults with PET evidence of tau in the absence of amyloid-beta demonstrate clinical, cognitive and biomarker characteristics that are consistent with groups traditionally considered to be on the Alzheimer’s disease continuum.

## Graphical Abstract

**Graphical abstract:**
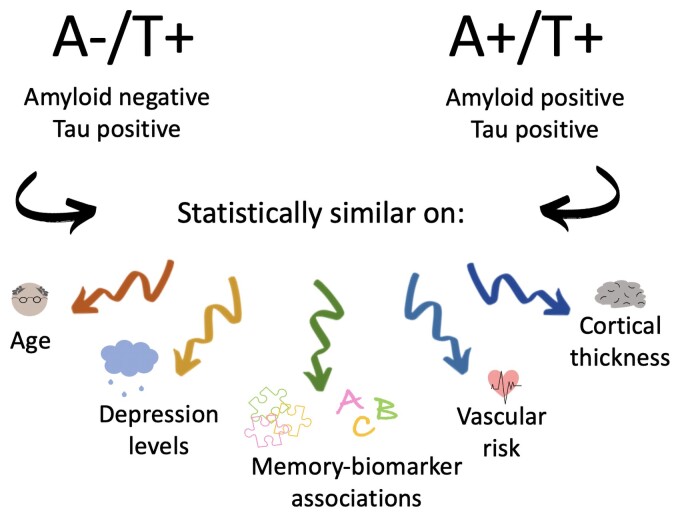

## Introduction

Alzheimer’s disease has historically been characterized by the presence of two pathologic proteins, amyloid-beta 1–42 (Aβ) and phosphorylated tau.^1^ Although these pathologies were initially given equal consideration in the definition of Alzheimer’s disease,^1^ more recent models of pathologic progression have proposed the presence of Aβ as the defining feature of Alzheimer’s disease.^2,3^ Indeed, the 2018 NIA-AA Alzheimer’s disease biological framework necessitates the presence of Aβ biomarkers for placement on the Alzheimer’s continuum, whereas tau biomarker positivity in the absence of Aβ is instead designated as ‘non-Alzheimer’s pathologic change’.^4^ Similarly, pathologic evidence of medial temporal tau in the absence of Aβ has been proposed as a construct separable from Alzheimer’s disease known as ‘primary age-related tauopathy’ or ‘PART’, in which these individuals typically have limited cognitive deficits, more advanced age and lower Alzheimer’s disease genetic risk relative to Alzheimer’s disease.^5^ Controversy remains, however, as to whether PART is truly an alternative pathologic process or whether it may reflect an early stage of the Alzheimer’s continuum.^6,7^

The nomenclature for the two discrepant Aβ and tau positivity groups, conversely labelled as ‘Alzheimer’s pathologic change’ (A+/T−) and ‘non-Alzheimer’s pathologic change’ (A−/T+),^4^ suggests that A+/T− individuals might be farther along in terms of cognitive and biomarker progression relative to A−/T+ individuals. Additionally, PART theory would suggest that, in terms of cognition, A−/T+ would look most similar to A−/T− whereas A+/T− would look most similar to A+/T+. Whereas the previous studies have compared the features of the A−/T+ group with the ‘biomarker normal’ (i.e. A−/T−) and ‘AD’ group (i.e. A+/T+), a direct comparison with the other discrepant group (i.e. A+/T−) may yield additional insight. Investigating the patterns of clinical, cognitive and biomarker characteristics that emerge across these groups is an important step in clarifying the nature of the A−/T+ profile in relation to Alzheimer’s disease.

In contrast to PART theory and the AT(N) framework, our prior preliminary study defined an A−/T+ group using cortical Aβ and medial temporal (i.e. Braak stage I/II) tau PET imaging and demonstrated that this group had intermediate cognitive performance between the biomarker normal (A−/T−) and Alzheimer’s disease (A+/T+) groups, suggesting the possibility that the A−/T+ group may represent an early stage on the Alzheimer’s disease continuum.^8^ Similarly, a recent study by Yoon *et al*.^9^ defined an A−/T+ group using cortical Aβ and medial temporal (i.e. Braak stage I/II) and/or inferolateral temporal (i.e. Braak stage III/IV) tau PET imaging and similarly found intermediate global cognitive deficits as well as intermediate hippocampal volumes relative to A−/T− and A+/T+ groups, concluding that this heterogeneous group may represent a combination of individuals with PART and individuals with Alzheimer’s disease.

Given increasing evidence that the A−/T+ biomarker group may be confluent with the Alzheimer’s disease continuum,^8,9^ more research is needed to comprehensively characterize this group. Specifically, examination of both objective and subjective cognitive measures across multiple domains may provide insight into the clinical stage that corresponds to these A/T biomarker stages. In this study, we will assess sensitive neuropsychological measures (i.e. objective cognition) and self-reported measures (i.e. subjective cognition) across the domains of memory, language and executive function. We hypothesize that the neuropsychological performance across all domains will follow the order of A−/T− > A−/T+ = A+/T− > A+/T+, where > indicates better performance. For subjective cognitive complaints across all domains, we hypothesize that A−/T− < A−/T+ = A+/T− < A+/T+, where < indicates fewer cognitive complaints.

In addition to investigating objective and subjective cognitive abilities, we will also characterize brain structure across these A/T groups. Specifically, we will examine cortical thickness across widespread regions of temporal, parietal and frontal cortex, as well as global white matter lesion burden as a brain-based index of vascular risk to assess whether any observed cognitive deficits may be attributable to vascular pathology. We hypothesize that cortical thickness across temporal regions will follow the pattern of A−/T− > A+/T− = A−/T + > A+/T+, where > indicates higher thickness. However, for parietal and frontal regions, we hypothesize that the A−/T+ group will be equivalent to the A−/T− and A+/T− groups, given the relatively circumscribed nature of tau pathology within temporal regions in this group, whereas the A+/T+ group will have lower thickness values due to their more widespread distribution of pathology. In terms of vascular risk, we hypothesize that vascular risk will be lowest in the A−/T− group and equal across other groups. Finally, we will assess for associations between objective memory scores and temporal cortical regions to investigate the presence and strength of biomarker–cognition associations across A/T groups. We predict that only A−/T+ and A+/T+ groups will have significant positive associations between memory and temporal cortical thickness.

This study will use PET imaging to designate A/T groups based on cortical Aβ and a meta-temporal tau region, with a particular focus on the A−/T+ or ‘PART’ group as it relates to the A+/T− and A+/T+ groups who are traditionally considered to be on the Alzheimer’s disease continuum. This A−/T+ group will be comprehensively characterized using demographic, clinical, neuropsychological, subjective cognition, vascular risk and cortical thickness variables. We expect that, in general, the A−/T+ group will look most similar to the A+/T− group on cognitive and biomarker variables.

## Materials and methods

### Study data

The data sets used in the preparation of this article were obtained from the Alzheimer’s Disease Neuroimaging Initiative (ADNI) database (adni.loni.usc.edu). The ADNI was launched in 2003 as a public–private partnership, led by Principal Investigator Michael W. Weiner, MD. The primary goal of ADNI has been to test whether serial MRI, PET, other biological markers and clinical and neuropsychological assessment can be combined to measure the progression of mild cognitive impairment (MCI) and early Alzheimer’s disease. For up-to-date information, see www.adni-info.org.^10^

### Participants

This study included 703 participants without dementia from ADNI who had concurrent Aβ PET, tau PET and demographic data. The data were accessed on 20 January 2022. All participants had concurrent subjective and cognitive complaint data. A subset of 660/703 participants had apolipoprotein E (APOE) ε4 data. A subset of 619/703 participants had concurrent complete neuropsychological data. A subset of 450/703 participants had concurrent MRI data with FreeSurfer parcellation. A subset of 265/703 participants had concurrent white matter hyperintensity imaging data. Participants at different clinical stages were included (438 cognitively unimpaired and 181 with MCI classified using neuropsychological criteria). Overall, the sample was predominately white (90%) and female (51%), with a mean (standard deviation) age of 73.3 (7.5) years and an education level of 16.4 (2.5) years.

### Demographic and clinical data

Demographic data included participant age, sex, level of education and race. Depression was assessed using the Geriatric Depression Scale (GDS). Notably, there was a limited range for this measure as participants were excluded from ADNI if they had a baseline GDS > 5.

### Cognitive data

#### Neuropsychological assessment

Neuropsychological measures were obtained across domains of memory [Auditory Verbal Learning Test (AVLT) delayed recall and recognition], language {animal fluency, confrontation naming [i.e. Boston Naming Test (BNT) or Multilingual Naming Test (MiNT)]} and executive function [Trail Making Test (TMT) parts A and B]. Note that participants either had the BNT or the MiNT as a measure of naming; these scores were converted to percent correct to place them on the same scale and create one single ‘naming’ measure. Z-scores were calculated for individual neuropsychological measures using predicted values relative to a robust normal control group (e.g. remained cognitively intact throughout the duration of their participation) that adjusted for age, sex and education level such that higher scores indicated better performance.

#### Diagnostic criteria

MCI was classified using comprehensive neuropsychological criteria based on the presence of (i) two impaired scores in one cognitive domain or (ii) one impaired score across all three cognitive domains.^11^ Demographically adjusted *z*-scores from the above neuropsychological measures were used for diagnostic classification. Participants who did not meet MCI criteria were considered cognitively unimpaired.

#### Subjective cognitive complaints

The Everyday Cognition (ECog) questionnaire assessed self-reported subjective cognitive complaints. Composite scores averaged across memory, language and executive function domains were assessed such that higher scores indicated worse subjective cognition.

### Biomarker data

#### PET imaging

Biomarkers of Aβ (Florbetapir or Florbetaben) and tau (Flortaucipir) were assessed using PET imaging. For Aβ PET, a cortical summary measure called a region of interest (ROI) was used that included regions vulnerable to early Aβ deposition.^12,13^ For tau PET, a composite meta-temporal ROI representative of early-to-moderate tau pathology was used that included the amygdala, entorhinal cortex, fusiform gyrus, inferior temporal gyrus and middle temporal gyrus.^14^ Standardized uptake value ratios (SUVRs) were calculated by dividing the SUV for each ROI by the whole cerebellum SUV (Aβ PET) or the inferior cerebellar grey SUV (tau PET). Tau PET values were not corrected for partial volume effects. The Aβ SUVR values were converted to a centiloid scale to standardize across the two PET tracers.^15^ Positivity thresholds were determined based on previously published cut-offs: Aβ positivity was defined as cortical summary centiloid > 28.8 to indicate established pathology (maximizing Youden’s index based on CSF Aβ/p-tau positive or negative),^16^ and tau positivity was defined as meta-temporal SUVR > 1.23 to indicate established pathology (maximizing accuracy based on younger controls or Aβ positive cognitively impaired older adults).^17^

#### Freesurfer imaging

Cortical thickness measurements for the entorhinal cortex, inferior temporal gyrus, precuneus and superior frontal gyrus were obtained from T_1_-weighted anatomical MRI processed cross-sectionally using FreeSurfer version 5.1 or 6.0 [ADNI files: UCSF—Cross-Sectional FreeSurfer (5.1); UCSF—Cross-Sectional FreeSurfer (6.0)].

These regions were chosen for their vulnerability to early Alzheimer’s disease pathology^18^ and to be consistent with regions included in the cortical summary Aβ PET and meta-temporal tau PET measures.

#### Apolipoprotein E

APOE positivity was defined by the presence of one or more ε4 alleles.

#### Vascular risk

The pulse pressure values were obtained from blood pressure measurements as an index of arterial stiffening, where pulse pressure = (systolic blood pressure − diastolic blood pressure)/systolic blood pressure. Modified Hachinski ischaemia scale (ADNI file: modified Hachinski ischaemia scale) scores were assessed for 10-year stroke risk with scores dichotomized as low risk (score = 0) or high risk (score > 0). White matter hyperintensity values were obtained from MRI data as a measure of cerebrovascular small vessel disease (ADNI file: UCD—white matter hyperintensity volumes).

### Statistical analysis

Participants were classified into one of the four groups based on cortical Aβ and meta-temporal tau PET positivity: A−/T−, A−/T+, A+/T− and A+/T+. Proportions of participants in each group were examined. Groups were compared on distribution of sex (male/female), race (White/non-White), clinical classification (cognitively unimpaired/MCI), APOE ε4 (negative/positive) and Hachinski risk level (low/high) using χ^2^tests for independence. Groups were compared on age and education level using ANOVAs. Groups were compared on depression level, Aβ PET levels and tau PET Braak meta-temporal ROI levels, pulse pressure, white matter hyperintensity levels and regional cortical thickness/volume using ANCOVAs adjusting for age, sex and APOE ε4 positivity. Groups were compared on neuropsychological performance and subjective cognitive complaints using ANCOVAs adjusting for age, sex, education and APOE ε4 positivity. Multiple linear regression models adjusting for age, sex, education and APOE ε4 positivity assessed for associations between memory scores (i.e. AVLT delayed recall and recognition) and associated cortical thickness regions (i.e. entorhinal and inferior temporal gyrus) across the sample and separately within A/T groups. Data with non-normal distributions underwent Box–Cox transformation (i.e. pulse pressure, white matter hyperintensity values, entorhinal cortex, AVLT recognition, naming, TMT A, TMT B and all subjective cognitive complaint domains) with the exception of GDS score due to the interval nature of this variable (see Supplementary Figs 1–3 for distributions before and after transformation). Data presented in tables include raw means to facilitate the interpretation of values, but it should be noted that model estimates reflect transformed and residualized variables. Figures include transformed and residualized outcome variables to depict group comparisons after adjusting for covariates. All statistical tests were two-tailed with an alpha = 0.05.

### Data availability

Data used in this study are available through the ADNI and can be found at http://adni.loni.usc.edu/.

## Results

**Figure 1:**
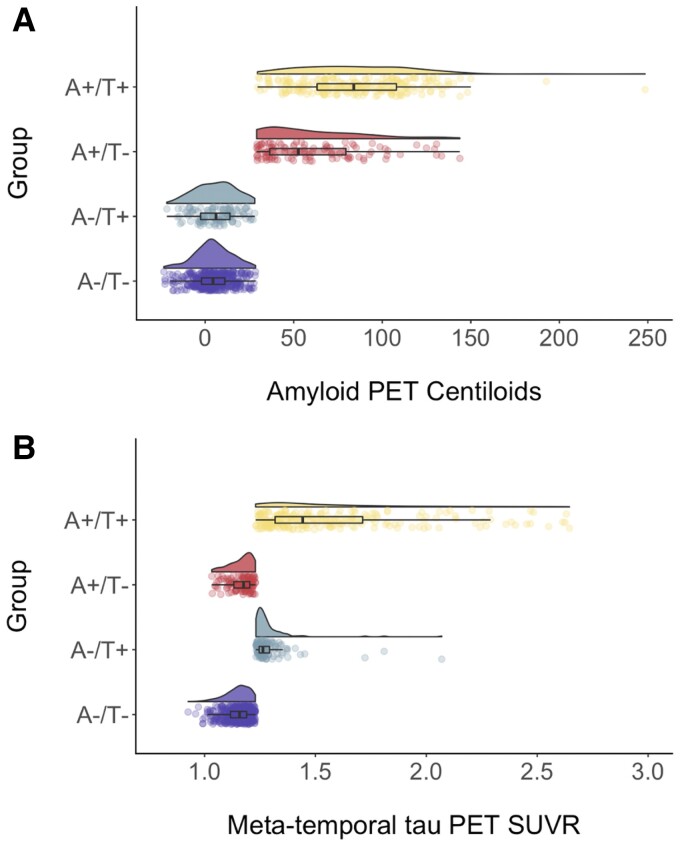
**A/T group distributions of Aβ PET centiloid and tau PET SUVR levels.** Raincloud plots depicting Aβ PET centiloid levels (**A**) and tau PET SUVR levels (**B**) across A−/T− (purple, bottom), A−/T + (blue–green, second from bottom), A+/T− (red, second from top) and A+/T+ (yellow, top)) groups. ANCOVAs indicated that groups differed on levels of Aβ PET centiloid levels [*F*(3,653) = 528.3, *P* < 0.001] an meta-temporal tau PET SUVR levels [*F*(3,653) = 153.3, *P* < 0.001].

Of the 703 participants in the sample, the largest portion was classified as A−/T− (47.23%). An additional 27.03% of participants were classified as A+/T+ and 12.23% were classified as A+/T−. Of particular interest is the A−/T+ group, which comprised 13.51% of the sample—a small but not insignificant proportion. As expected, these groups differed on levels of meta-temporal tau PET [*F*(3,653) = 153.3, *P* < 0.001], such that the T− groups were not statistically different (A−/T− median = 1.15; A+/T− median = 1.16), the A−/T+ group had significantly higher tau than the T− groups (median = 1.29) and the A+/T+ group had significantly higher tau than all other groups (median = 1.59). Groups also differed on levels of cortical Aβ PET [*F*(3,653 = 528.3, *P* < 0.001] such that the A− groups were not statistically different (A−/T− median = 4.79; A−/T+ median = 6.14), the A+/T− group had significantly higher Aβ than the A− groups (median = 51.58) and the A+/T+ group had significantly higher Aβ than all other groups (median = 83.98). See Table 1 and Fig. 1 for the distributions of Aβ and tau PET across the A/T groups.

**Table 1:** Comparison of A/T groups on demographic variables and PET values

Variable	A−/T−	A−/T+	A+/T−	A+/T+	Test statistic	*P*-value
*n* (% of sample)	332 (47.2%)	95 (13.5%)	86 (12.2%)	190 (27.0%)	N/A	N/A
Age, mean (SD)	71.5 (7.3)	74.7 (6.7)^a^	75.5 (8.2)^a^	74.7 (7.2)^a^	*F* = 12.6	<0.001
Sex, % Female	53.3%	47.4%	40.0%	54.5%	χ^2^ = 6.6	0.08
Education, mean (SD)	16.5 (2.5)	17.2 (2.2)^a^	16.7 (2.5)	15.9 (2.5)^c^	*F* = 6.9	<0.001
Race, % White	88.7%	86.7%	90.6%	91.6%	χ^2^ = 2.3	0.83
GDS score, mean (SD)	1.1 (1.4)	1.6 (2.1)	1.1 (1.2)	1.6 (1.7)^b^	*F* = 4.7	0.002
Aβ centiloid, median (SD)	4.8 (10.4)	6.1 (11.4)	51.6 (29.5)^a^	84.0 (29.0)^c^	*F* = 528.3	<0.001
Tau SUVR, median (SD)	1.2 (0.1)	1.3 (0.1)^a^	1.2 (0.1)	1.6 (0.4)^c^	*F* = 153.3	0.001

GDS, geriatric depression scale; *n,* sample size; SD, standard deviation.

^a^ Significantly different from the A−/T− group.

^b^ Significantly different from the A−/T− and A+/T− groups.

^c^ Significantly different from all other groups.

Demographic characteristics across these A/T groups are presented in Table 1. Groups significantly differed in age [*F*(3,699) = 12.6, *P* < 0.001] such that the A−/T− group was significantly younger than all other groups, who did not differ from one another; education levels [*F*(3,700) = 6.9, *P* < 0.001] such that the A+/T+ group had significantly lower education levels than all other groups and the A−/T+ group had significantly higher education levels than the A−/T− group; and depression levels [*F*(3,651) = 4.7, *P* = 0.002] such that the A+/T+ group had significantly higher GDS scores than the A−/T− and A+/T− groups, but not the A−/T+ group. Groups did not significantly differ on race distribution (*P* = 0.83) or sex distribution (*P* = 0.08).

**Figure 2:**
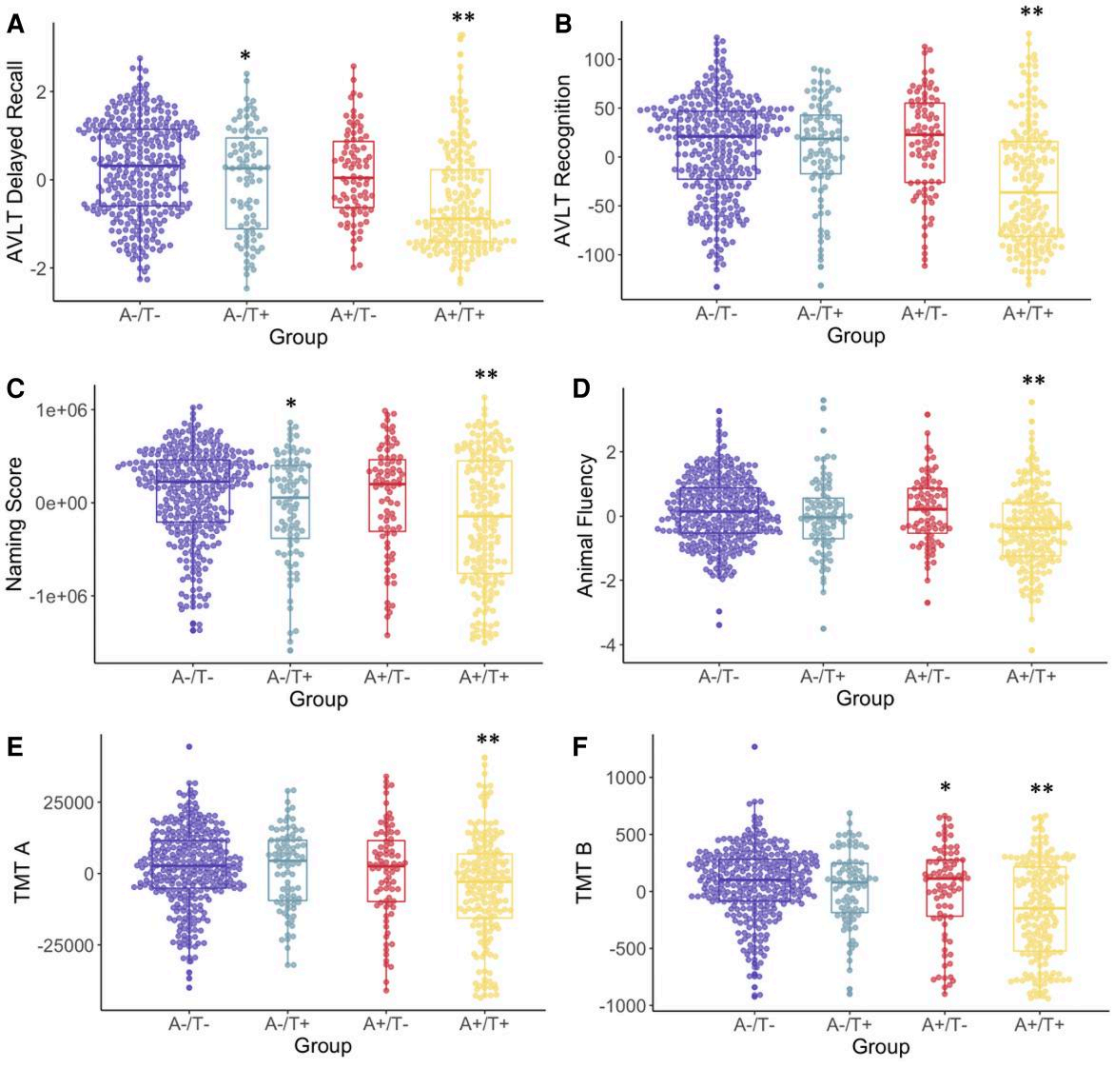
**A/T group differences in neuropsychological scores.** Neuropsychological scores (**A**, AVLT delayed recall; **B**, AVLT recognition; **C**, animal fluency; **D**, naming score; **E**, TMT A; **F**, TMT B) across A/T groups. Transformed and residualized data are depicted. ANCOVAs indicated a significant difference between groups on all measures: AVLT delayed recall [*F*(3,650) = 19.5, *P* < 0.001], AVLT recognition [*F*(3,648) = 25.2, *P* < 0.001], naming [*F*(3, 647) = 10.0, *P* < 0.001], animal fluency [*F*(3,651) = 11.3, *P* < 0.001], TMT A [*F*(3,647) = 8.9, *P* < 0.001] and TMT B [*F*(3,638) = 17.8, *P* < 0.001]. *Statistically different from the A−/T− group. **Significantly different from all other groups.

When examining clinical classification (i.e. cognitively unimpaired or MCI), there was a significant difference across groups (χ^2^ = 39.3, *P* < 0.001) with the highest rate of MCI in the A+/T+ group (50.8%) and similar rates across the other groups (22.0–29.5%). Groups significantly differed on all neuropsychological measures: AVLT delayed recall [*F*(3,650) = 19.5, *P* < 0.001], AVLT recognition [*F*(3,648) = 25.2, *P* < 0.001], naming [*F*(3,647) = 10.0, *P* < 0.001], animal fluency [*F*(3,651) = 11.3, *P* < 0.001], TMT A [*F*(3,647) = 8.9, *P* < 0.001] and TMT B [*F*(3,638) = 17.8, *P* < 0.001]. On AVLT delayed recall and naming, the A+/T+ group had lower scores than all other groups and the A−/T+ group had lower scores than the A−/T− group. On TMT B, the A+/T+ group had lower scores than all other groups and the A+/T− group had lower scores than the A−/T− group. On AVLT recognition, animal fluency and TMT A, the A+/T+ group had lower scores than all other groups, who did not differ from one another. See Table 2 and Fig. 2 for more information.

**Table 2:** Comparison of A/T groups on neuropsychological z-scores and ECog questionnaire domain scores

Variable	A−/T−	A−/T+	A+/T−	A+/T+	Test statistic	*P*-value
Diagnosis, % MCI	22.0%	23.3%	29.5%	50.8%^d^	χ^2^ = 39.3	<0.001
AVLT recall, mean (SD)	−0.3 (1.1)	−0.6 (1.2)^a^	−0.6 (1.0)	−1.2 (1.1)^d^	*F* = 19.5	<0.001
AVLT recognition, mean (SD)	−0.4 (1.2)	−0.5 (1.3)	−0.6 (1.4)	−1.9 (1.9)^d^	*F* = 25.2	<0.001
Naming, mean (SD)	0.0 (1.1)	−0.4 (1.5)^a^	−0.4 (1.4)	−1.1 (2.4)^d^	*F* = 10.0	<0.001
Animal fluency, mean (SD)	−0.1 (1.0)	−0.3 (1.1)	−0.2 (1.1)	−0.8 (1.2)^d^	*F* = 11.3	<0.001
TMT A, mean (SD)	−0.1 (1.1)	−0.1 (1.0)	−0.3 (1.4)	−1.1 (2.5)^d^	*F* = 8.9	<0.001
TMT B, mean (SD)	−0.1 (1.2)	−0.2 (1.2)	−0.6 (1.8)^a^	−1.5 (2.4)^d^	*F* = 17.8	<0.001
ECog memory, mean (SD)	1.8 (0.7)	1.9 (0.7)	2.0 (0.8)^b^	2.4 (0.9)^d^	*F* = 14.0	<0.001
ECog language, mean (SD)	1.6 (0.6)	1.6 (0.7)	1.8 (0.6)^a^	1.9 (1.0)^c^	*F* = 6.1	<0.001
ECog executive, mean (SD)	1.5 (0.6)	1.5 (0.6)	1.6 (0.5)	1.9 (1.0)^d^	*F* = 8.8	<0.001

AVLT, auditory verbal learning test; TMT, trail making test; SD, standard deviation. Note that the values reported here do not reflect the transformed and residualized values used in model estimates.

^a^ Significantly different from the A−/T− group.

^b^ Significantly different from the A−/T + group.

^c^ Significantly different from the A−/T− and A−/T+ groups.

^d^ Significantly different from all other groups.

**Figure 3:**
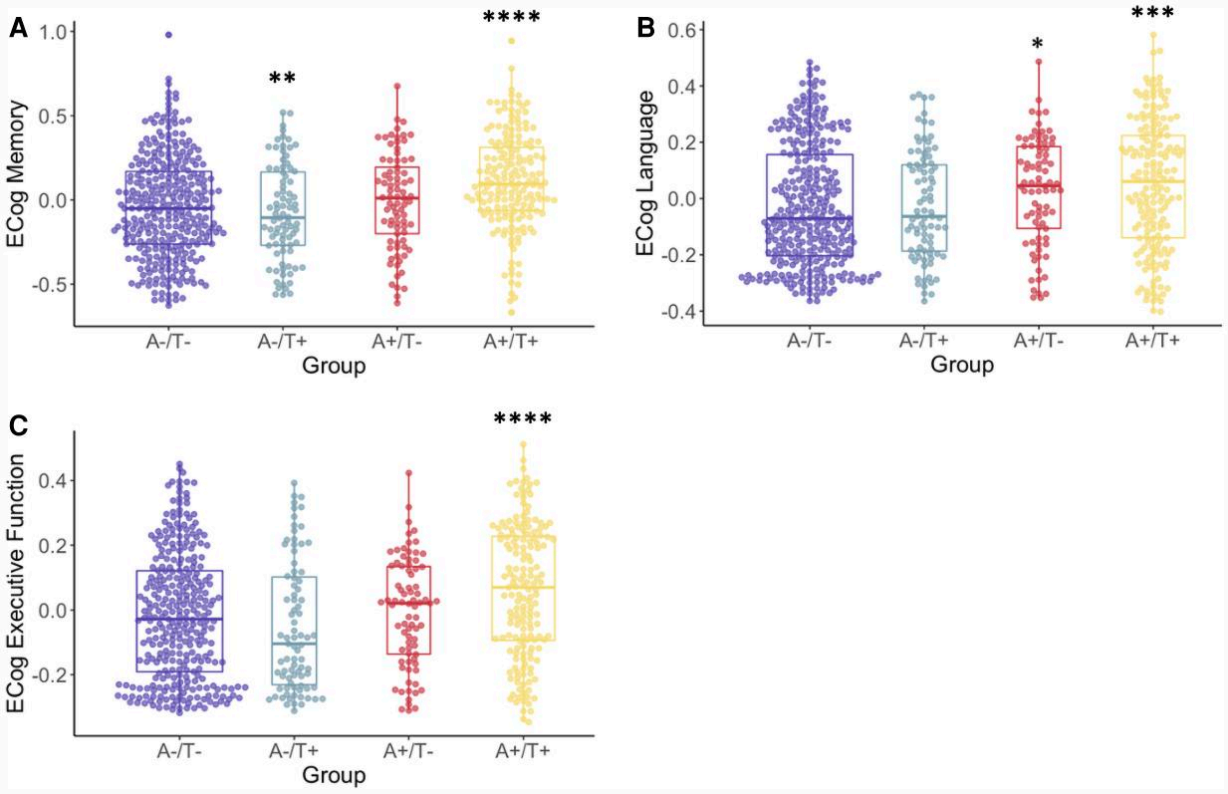
**A/T group differences in ECog questionnaire scores.** ECog scores (**A,** memory; **B**, language; **C**, executive function) across A/T groups. Transformed and residualized data are depicted. ANCOVAs indicated a significant difference between groups on all measures: Memory [*F*(3,646) = 14.0, *P* < 0.001], language [*F*(3,642) = 6.1, *P* < 0.001] and executive function [*F*(3,634) = 8.8, *P* < 0.001]. *Significantly different from the A−/T− group. **Significantly different from the A−/T+ group. ***Significantly different from the A−/T− and A−/T+ groups. ****Significantly different from all other groups.

Groups significantly differed on all domains of subjective cognitive complaints: memory [*F*(3,646) = 14.0, *P* < 0.001], language [*F*(3,642) = 6.1, *P* < 0.001] and executive function [*F*(3,634) = 8.8, *P* < 0.001]. For subjective memory, the A+/T+ group had higher scores (i.e. worse subjective memory) than all other groups and the A+/T− group had higher scores than the A−/T+ group. For subjective language, the A+/T+ group had higher scores (i.e. worse subjective language) than the A−/T− and A−/T+ groups and the A+/T− group had higher scores than the A−/T− group. For subjective executive function, the A+/T+ group (i.e. worse subjective executive function) than all other groups, who did not differ from one another. See Table 3 and Fig. 3 for more information.

**Table 3:** Comparison of A/T groups on cortical thickness, vascular risk and APOE status

Variable	A−/T−	A−/T+	A+/T−	A+/T+	Test statistic	*P*-value
EC thickness, mean (SD)	3.58 (0.4)	3.37 (0.6)^a^	3.58 (0.3)	3.24 (0.5)^a^	*F* = 12.6	<0.001
ITG thickness, mean (SD)	2.86 (0.1)	2.83 (0.2)	2.87 (0.1)	2.76 (0.2)^a^	*F* = 5.6	<0.001
Precuneus thickness, mean (SD)	2.34 (0.1)	2.31 (0.1)	2.30 (0.2)	2.23 (0.2)^a^	*F* = 5.3	0.001
SFG thickness, mean (SD)	2.56 (0.1)	2.52 (0.1)	2.51 (0.2)	2.51 (0.1)	*F* = 1.2	0.31
Pulse pressure, mean (SD)	2.00 (0.1)	2.00 (0.1)	2.01 (0.1)	2.01 (0.1)	*F* = 0.8	0.45
HIS score % high risk	45.2%	54.2%	50.0%	50.7%	*F* = 2.2	0.54
Global WMH, mean (SD)	5.39 (9.1)	6.19 (7.9)	11.86 (16.7)	10.3 (17.1)	χ^2^ = 0.6	0.59
APOE ε4 status, % ε4 positive	23.3%	18.6%	51.0%^b^	64.8%^c^	χ^2^ = 116.2	<0.001

EC, entorhinal cortex; HIS, Hachinski ischaemic scale; ITG, inferior temporal gyrus; SD, standard deviation; SFG, superior frontal gyrus; WMH, white matter hyperintensities.

Note that the values reported here do not reflect the transformed and residualized values used in model estimates.

^a^ Significantly different from the A−/T− and A+/T− groups.

^b^ Significantly different from the A−/T− and A−/T + groups.

^c^ Significantly different from all other groups.

**Figure 4:**
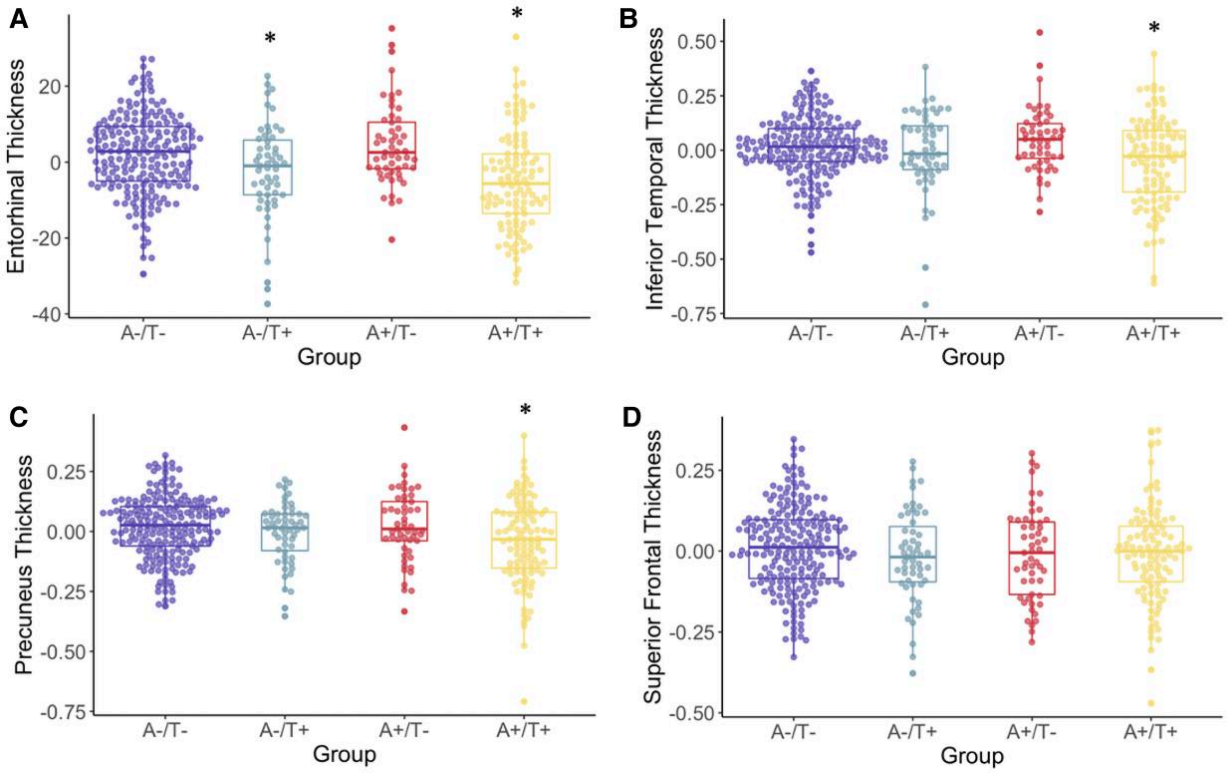
**A/T group differences in cortical thickness.** Cortical thickness profiles (**A**, entorhinal; **B**, inferior temporal; **C**, precuneus; **D**, superior frontal) across A/T groups. Transformed and residualized data are depicted. ANCOVAs indicated a significant difference between groups for the entorhinal cortex [*F*(3,412) = 12.6 *P* < 0.001], the inferior temporal gyrus [*F*(3,397) = 5.6, *P* < 0.001] and the precuneus [*F*(3,412) = 5.3, *P* = 0.001]. Groups did not significantly differ for the superior frontal gyrus. *Statistically different from the A−/T− and A+/T− groups.

Groups were compared on regional cortical thickness of the entorhinal cortex, inferior temporal gyrus, precuneus and superior frontal gyrus. For the entorhinal cortex, there was a significant difference across groups [*F*(3,412) = 12.6, *P* < 0.001] such that the A−/T+ and A+/T+ groups, who did not differ from each other, had lower cortical thickness than the A−/T− and A+/T− groups, who did not differ from each other. For the inferior temporal gyrus, there was a significant difference across groups [*F*(3,397) = 5.6, *P* < 0.001] such that the A+/T + group had lower cortical thickness than the A−/T− and A+/T− groups, but not the A−/T+ group. For the precuneus, there was a significant difference across groups [*F*(3,412) = 5.3, *P* = 0.001] such that the A+/T+ group had lower cortical thickness than the A−/T− and A+/T− groups, but not the A−/T+ group. For the superior frontal gyrus, there was no significant difference across groups [*F*(3,409) = 1.2, *P* = 0.31]. See Table 3 and Fig. 4 for more information.

When comparing groups on APOE ε4 positivity rates, there was a significant difference across groups (χ^2^ = 116.2, *P* < 0.001) with the lowest positivity rate observed in the A−/T− (23.3% ε4+) and A−/T+ (18.6% ε4+) groups, followed by the A+/T− group (51.0%) and the highest rates observed in the A+/T+ group (64.8%).

Groups were compared on several indices of vascular risk and did not differ on pulse pressure [*F*(3,648) = 0.8, *P* = 0.45], global white matter hyperintensity values [*F*(3,176) = 0.6, *P* = 0.59] or Hachinski score (χ^2^ = 2.2, *P* = 0.54).

**Figure 5:**
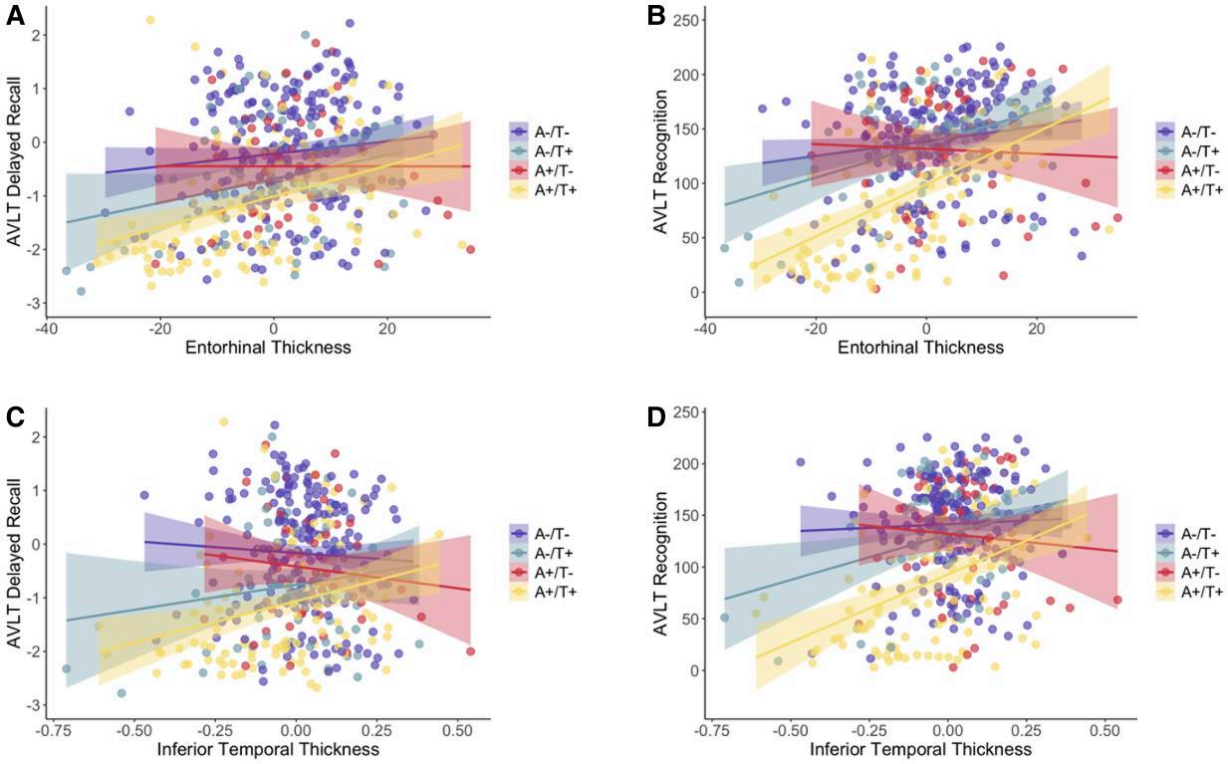
**A/T group associations between cortical thickness and neuropsychological scores.** Scatterplots depicting AVLT delayed recall (**A**, **C**) and recognition **(B**, **D**) associations with entorhinal (**A**, **B**) and inferior temporal (**C**, **D**) cortical thickness across A/T groups. Transformed and residualized data are depicted. (**A**) AVLT delayed recall was significantly associated with entorhinal thickness only in the A+/T+ group (*t* = 3.5, *P* = 0.001). (**B**) AVLT recognition was significantly associated with entorhinal thickness in the A−/T− (*t* = 2.3, *P* = 0.02), A−/T+ (*t* = 2.9, *P* = 0.006) and A+/T+ (*t* = 6.3, *P* < 0.001) groups but not the A+/T− group. (**C**) AVLT delayed recall was significantly associated with inferior temporal thickness only in the A+/T+ group (*t* = 2.8, *P* = 0.01). (D) AVLT recognition was significantly associated with inferior temporal thickness in the A−/T+ (*t* = 2.4, *P* = 0.02) and A+/T+ (*t* = 4.8, *P* < 0.001) groups but not the A−/T− or A+/T− group.

Both AVLT delayed recall and recognition scores were significantly associated with entorhinal cortical thickness across the entire sample (recall *t* = 5.5, *P* < 0.001; recognition *t* = 7.5, *P* < 0.001). Within A/T groups, AVLT delayed recall was significantly associated with entorhinal thickness only in the A+/T+ group (*t* = 3.5, *P* = 0.001), whereas AVLT recognition was significantly associated with entorhinal thickness in the A−/T− (*t* = 2.3, *P* = 0.02), A−/T+ (*t* = 2.9, *P* = 0.006) and A+/T+ (*t* = 6.3, *P* < 0.001) groups but not the A+/T− group. Both AVLT delayed recall and recognition scores were also significantly associated with inferior temporal thickness across the entire sample (recall *t* = 2.6, *P* = 0.01; recognition *t* = 5.4, *P* < 0.001). Within A/T groups, AVLT delayed recall was significantly associated with inferior temporal thickness only in the A+/T + group (*t* = 2.8, *P* = 0.01), whereas AVLT recognition was significantly associated with inferior temporal thickness in the A−/T+ (*t* = 2.4, *P* = 0.02) and A+/T+ (*t* = 4.8, *P* < 0.001) groups but not the A−/T− or A+/T− group. See Fig. 5 for depictions of these associations.

## Discussion

Our study identified a group of individuals comprising 13.5% of an ADNI older adult sample that fell into the biomarker category of meta-temporal tau PET positivity in the context of Aβ PET negativity, which has historically been referred to as ‘PART’ or ‘non-Alzheimer’s pathologic change’.^4,5^ Notably, however, this A−/T+ group exhibited a profile of clinical and biomarker features that was statistically similar to the A+/T+ (i.e. ‘Alzheimer’s disease’) group; specifically, these groups had similar levels of depression, vascular risk and cortical thickness across temporal and parietal regions. Although the A+/T+ group had a worse neuropsychological performance than the A−/T+ group, it should be noted that the A−/T+ group demonstrated worse delayed recall and naming than the A−/T− group, suggesting intermediate cognitive deficits. Additionally, this group demonstrated associations between memory and medial/inferior temporal thickness that was not observed in the A+/T− group. Our results extend prior studies examining this A−/T+ group in ADNI^8,9^ to suggest that their neuropsychological and neurodegenerative profiles may be indicative of a potential Alzheimer’s disease pathologic process. Interestingly, our study demonstrated that the A−/T+ group may have marginally fewer subjective cognitive complaints in domains of memory and language relative to the A+/T− group, which, in the context of poorer neuropsychological performance in the A−/T+ group relative to the A−/T− group, may reflect a degree of anosognosia often seen in the MCI stage.^19,20^ Taken together, these findings suggest that the A−/T+ biomarker designation should be given equal attention as A+/T− and A+/T+ groups, who are more often included in research studies and clinical trials.

Questions may remain as to whether the A−/T+ group represents a stage on the Alzheimer’s disease continuum or that of a separate pathologic process (e.g. PART). There is a possibility that the tau PET tracer used (Flortaucipir) may have been binding to different tau isoforms or other pathologies,^21,22^ in which case this group may be better explained as an alternate pathologic process separable from Alzheimer’s disease, or otherwise as an atypical Alzheimer’s disease variant. Notably, however, even in the absence of high levels of Aβ, the A−/T+ group exhibited pathognomonic features characteristic of Alzheimer’s disease including specific deficits in memory recall and object naming, both common early cognitive features of Alzheimer’s disease^23^ and lower cortical thickness in the entorhinal cortex, a region affected early in Alzheimer’s disease as well as PART.^18^ The relative sparing of other cognitive scores in the context of lower cortical thickness across Alzheimer’s disease signature regions may be explained by a higher degree of cognitive reserve in the A−/T+ group, given their lower APOE genetic risk and a marginally higher level of education. However, our results did demonstrate an association between memory performance and temporal regions within the A−/T+ group, suggesting that the lower cortical thickness may indicate early neurodegeneration that is impacting memory. Research examining the longitudinal trajectories of this A−/T+ group is necessary to determine if they follow a declining trajectory across cognitive domains and cortical regions, as well as a transition from A−/T+ to A+/T+, which would suggest they may be on the Alzheimer’s disease continuum despite some initial cognitive resilience. Such a longitudinal examination is not possible at this time in the current data set due to low power across cells at follow-up visits but is a planned future direction of the current study when more data become available. It should be noted that individuals with PART may also demonstrate circumscribed cognitive deficits and medial temporal lobe atrophy as demonstrated by our findings;^7^ therefore, until longitudinal trajectories of these groups and pathologic confirmation are available, it remains unclear whether the A−/T+ group represents an early stage on the Alzheimer’s disease continuum, PART, an atypical Alzheimer’s disease variant, or another pathologic process.

Interestingly, a recent study also conducted in ADNI identified four distinct profiles of tau deposition and associated cognitive deficits to demonstrate atypical Alzheimer’s disease presentations among individuals who are primarily A+.^24^ Most notably, one of these four profiles comprising 19% of their sample was somewhat similar to our A−/T+ group with prominent lateral temporal tau deposition (in addition to MTL deposition) and specific deficits in language, although it should be noted that, unlike our A−/T+ group, the vast majority of individuals with this tau profile were Aβ positive. Vogel *et al*.^24^ also characterized their tau profiles longitudinally and demonstrated that the lateral temporal profile had the highest rate of global cognitive decline over time and eventual extension of tau deposition to parietal and frontal regions, suggesting that this profile may be on an Alzheimer’s disease trajectory. This recent study highlights the notion that Alzheimer’s disease pathologic and clinical presentations are heterogeneous and may not always progress through a singular prescribed series of stages. The emergence of an A−/T+ group in our study with certain ‘AD-like’ characteristics (i.e. poor recall and naming, entorhinal cortical thinning) and other dissimilar features (i.e. low APOE ε4 allele frequency) highlights the possibility that there may exist ‘non-traditional’ (i.e. initially Aβ negative) pathways to Alzheimer’s disease.

The debate as to whether the A−/T+ group reflects a non-Alzheimer’s pathologic process or an Alzheimer’s disease variant ultimately comes down to the nosology of AD. From a purely pathologic perspective, if one considers the presence of Aβ not only sufficient but also necessary in the definition of Alzheimer’s disease, that would negate the possibility of an A−/T+ subtype to be characterized as Alzheimer’s disease unless and until they progress to A+/T+. This reductionist view of Alzheimer’s disease, however, fails to consider the cognitive and clinical syndromal contributions to the definition of Alzheimer’s disease. Ultimately, the presence or absence of Aβ is inconsequential if there is no manifestation of cognitive and clinical deficits. This argument has recently been borne out in the FDA approval of aducanumab, a disease-modifying Alzheimer’s disease treatment that has largely failed to impart any clinical benefit despite its success in targeted reductions of Aβ.^25^ The questions as to whether aducanumab is truly ‘disease-modifying’ again depend on the nosological consideration of what it means to have Alzheimer’s disease and whether we consider it to be a purely pathologic construct or a combinatorial pathologic-syndromal construct. The International Working Group on the diagnosis of Alzheimer’s disease recommended that its diagnosis ‘be restricted to people who have positive biomarkers together with specific Alzheimer’s disease phenotypes, whereas biomarker positive cognitively unimpaired individuals should be considered only at-risk for progression to Alzheimer’s disease’.^26^ The results of our study argue that the incorporation of cognitive and clinical features into the definition of Alzheimer’s disease is critical, even as the field has shifted towards a biological perspective that largely ignores the ‘C’ (i.e. cognitive/clinical) in the AT(N)X classification system.^27^ However, regardless of the classification of this A−/T+ group as a non-Alzheimer’s process or as a stage on the Alzheimer’s disease continuum, the exclusion of this sizeable contingent of A−/T+ individuals from Alzheimer’s disease research will only perpetuate their exclusion from theoretical models of Alzheimer’s disease and reinforce the limited perspective on what is and is not Alzheimer’s disease.

A significant limitation of this study is the highly homogenous nature of the ADNI sample in terms of race/ethnicity, educational attainment, psychiatric well-being and overall physical health (including limited vascular risk). The limited variance in these characteristics makes it difficult to (i) ascertain group differences and (ii) generalize findings from a non-representative sample to the overall older adult population. Neuropathologic studies have demonstrated significant differences in pathologic profiles across racial/ethnic groups as well as a general likelihood of polypathology beyond Aβ and tau,^28,29^ patterns that were not observable in our study design and sample. An extension of our study with a more representative sample and inclusion of other pathologic markers (e.g. TDP-43, Lewy body) would help to further characterize our A−/T+ group and identify additional non-traditional pathologic profiles. Additionally, the use of different PET cut-points may have yielded different proportions of A/T groups. That said, the cut-points used in this study were selected based on well-established and validated methods previously reported in the literature. Further, an examination of Fig. 1 demonstrates there was a notable distribution of Aβ values in the A−/T+ group rather than a ‘cluster’ of subthreshold Aβ values near the cut-point. Also, statistically similar median Aβ values for the A−/T− and A−/T + groups suggest that subthreshold Aβ was likely not driving any differences in outcome variables. Finally, the cross-sectional nature of our study limits inferences about the progression of the A−/T+ group over time, including whether this group later becomes Aβ positive or demonstrates longitudinal cognitive decline and cortical thinning. Demonstration of these longitudinal features would bolster the notion that this group may belong to the Alzheimer’s disease continuum.

The notion that Alzheimer’s disease is synonymous with Aβ has come under scrutiny, given the failure of Aβ-modifying treatments to slow cognitive and clinical decline,^25,30,31^ and research increasingly suggests the need for a shift in theoretical perspective that considers other pathologic processes and drug targets. Our findings highlight the importance of investigating non-traditional pathways to Alzheimer’s disease and the inclusion of individuals with tau biomarker positivity in the absence of Aβ biomarker positivity in studies of Alzheimer’s disease. Consideration and further characterization of these A−/T+ individuals are critical to advance our understanding of the heterogeneous nature of Alzheimer’s disease, including its neuropathologic substrates and clinical manifestations.

## Supplementary Material

fcac272_Supplementary_Data

## Abbreviations

A =: amyloid
Aβ =: amyloid-beta 1–42
ADNI =: Alzheimer’s disease neuroimaging initiative
APOE =: apolipoprotein E
AVLT =: auditory verbal learning test
BNT =: Boston Naming Test
ECog =: Everyday Cognition
GDS =: Geriatric Depression Scale
MCI =: mild cognitive impairment
MiNT =: Multilingual Naming Test
PART =: primary age-related tauopathy
T =: tau
TMT =: Trail Making Test
