# Correction to: Comprehensive characterization of elevated tau PET signal in the absence of amyloid-beta

**DOI:** 10.1093/braincomms/fcad012

**Published:** 2023-01-24

This is a correction to: Alexandra J Weigand, Lauren Edwards, Kelsey R Thomas, Katherine J Bangen, Mark W Bondi, for the Alzheimer’s Disease Neuroimaging Initiative, Comprehensive characterization of elevated tau PET signal in the absence of amyloid-beta, *Brain Communications*, Volume 4, Issue 6, 2022, fcac272, https://doi.org/10.1093/braincomms/fcac272

In the originally published version of this manuscript author Lauren Edwards’s name was incorrectly published as “Lauren E Edwards.”

This error has been corrected.

