# Supplementary material for: Comprehensive characterization of elevated tau PET signal in the absence of amyloid-beta
Source: Brain Commun. 2022 Oct 26;4(6):fcac272. doi: 10.1093/braincomms/fcac272 (PMC9651027; doi:10.1093/braincomms/fcac272)
Supplement: fcac272_Supplementary_Data [file fcac272_supplementary_data.docx]

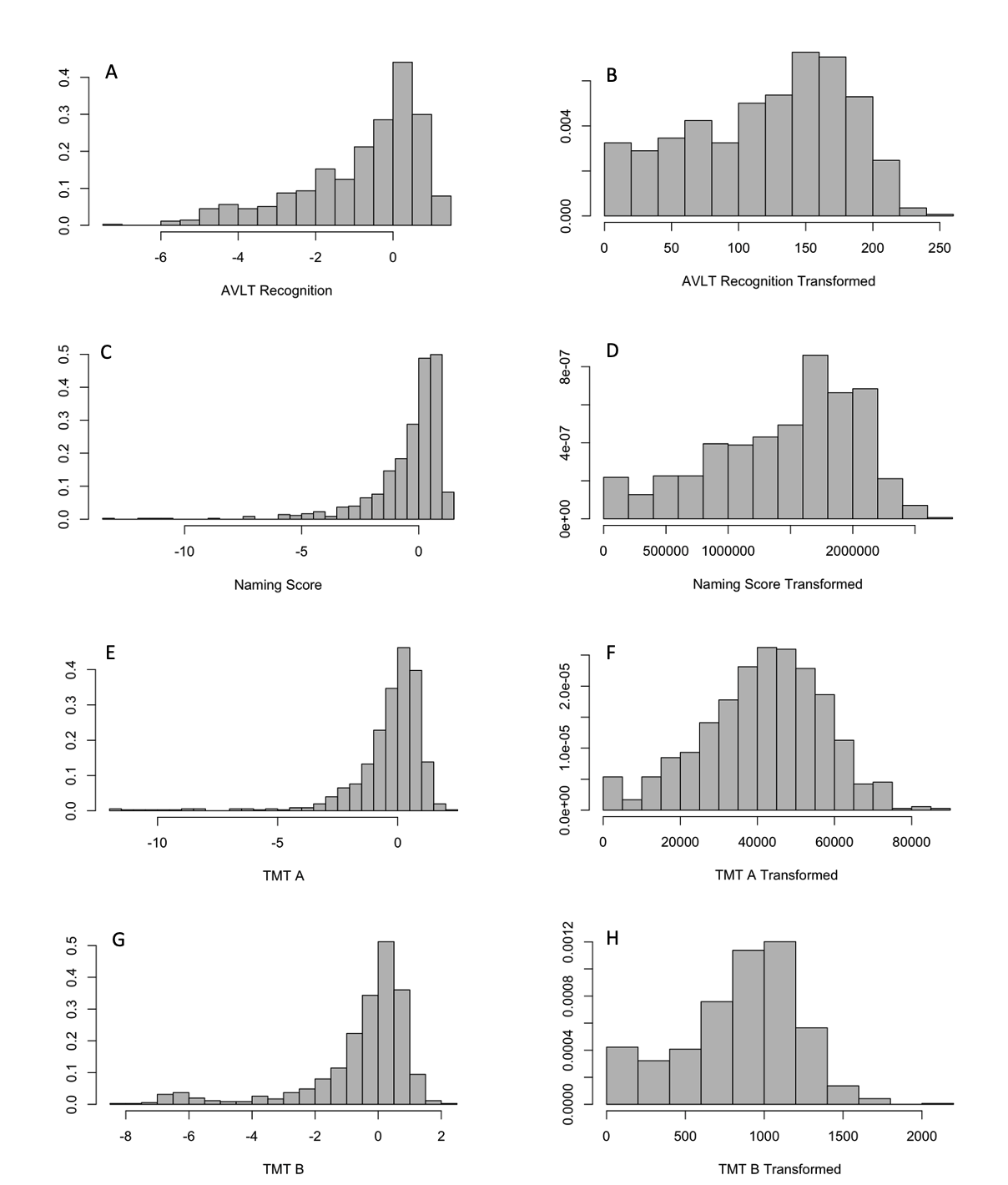


Supplementary Figure 1. Histograms of untransformed (A,C,E,G) and Box-Cox transformed (B,D,F,H) neuropsychological values (AVLT recognition: A,B; naming score: C,D; TMT A: E,F; TMT B: G,H). AVLT = Auditory Verbal Learning Test. TMT = Trail Making Test.


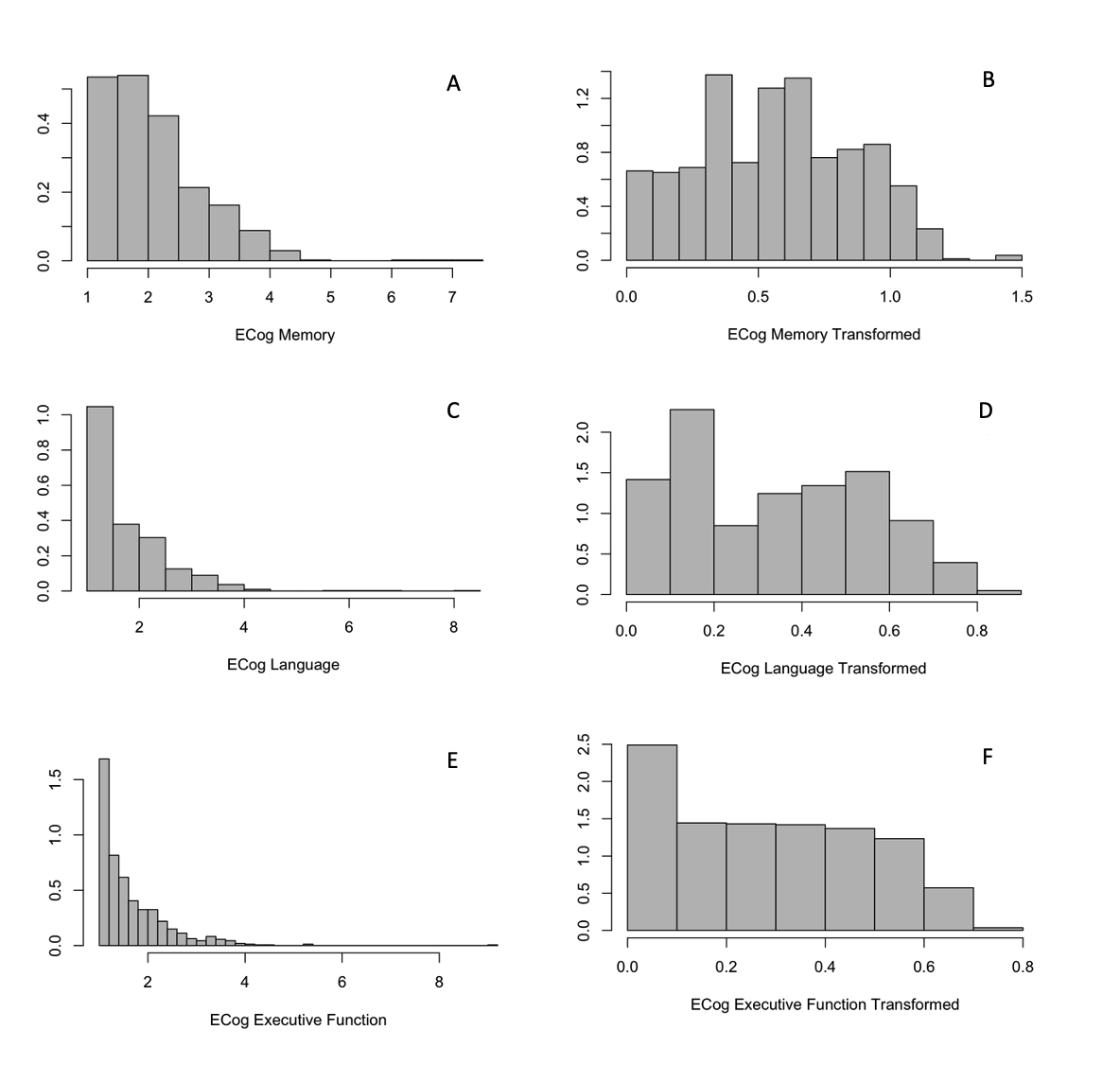

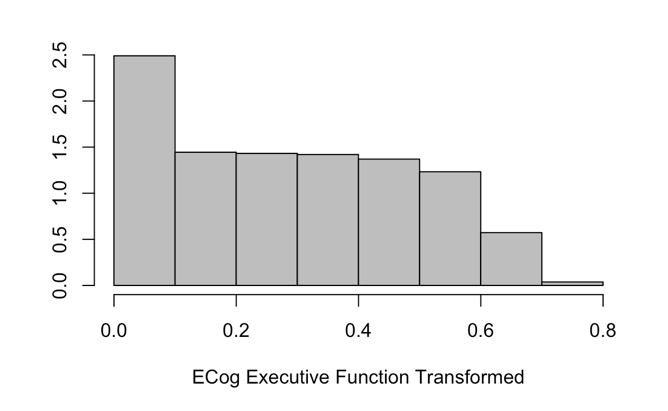


E

F

B

Supplementary Figure 2. Histograms of untransformed (A,C,E) and Box-Cox transformed (B,D,F) Everyday Cognition (ECog) questionnaire values (Memory: A,B; Language: C,D; Executive Function: E,F).


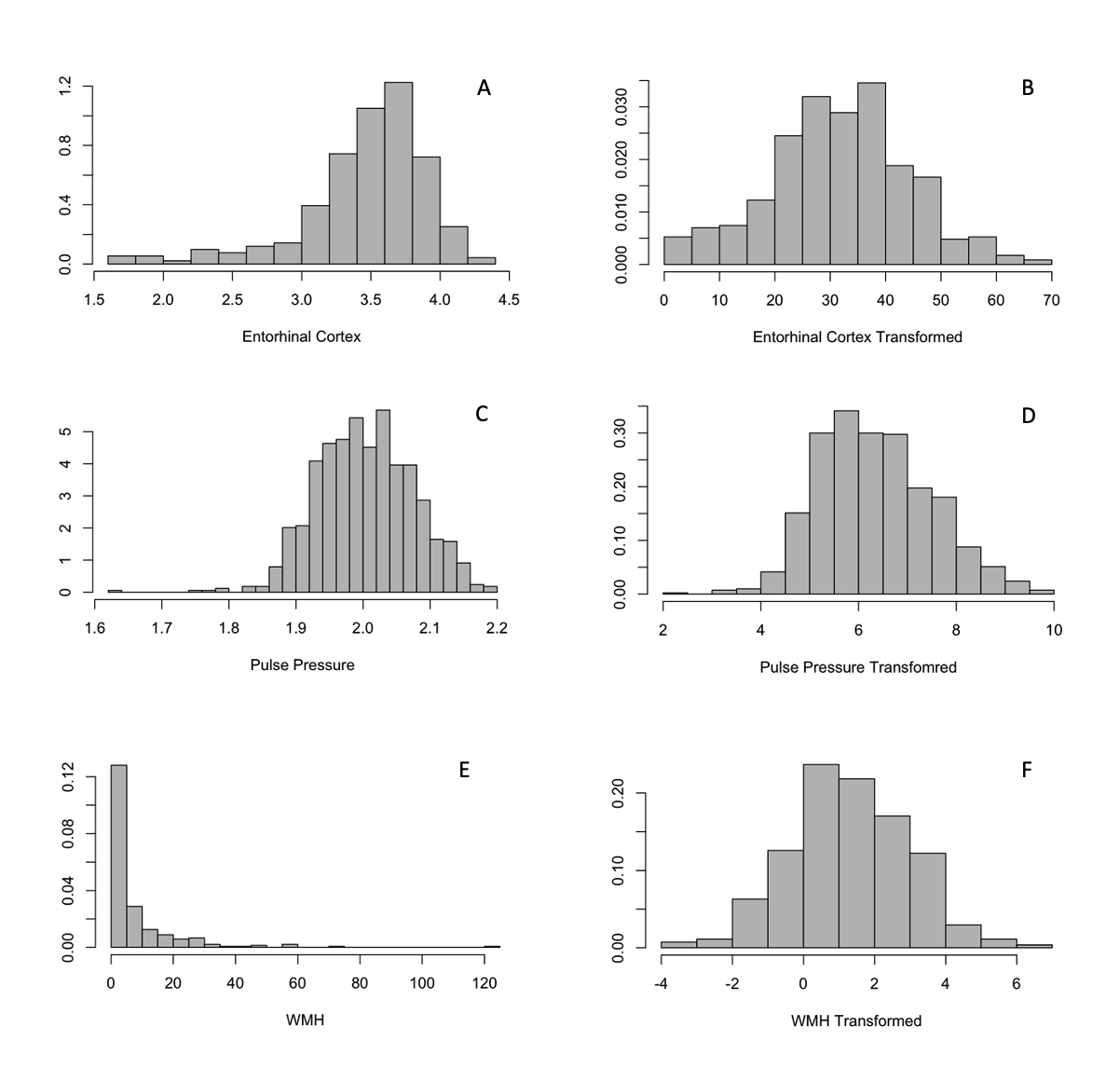

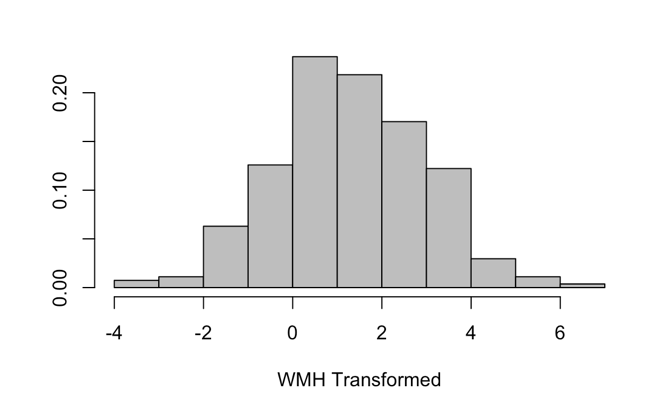


F

E

Supplementary Figure 3. Histograms of untransformed (A,C,E) and Box-Cox transformed (B,D,F) biomarker values (entorhinal cortex: A,B; pulse pressure: C,D; WMH: E,F). WMH = white matter hyperintensities.
